# The BAR Score Predicts and Stratifies Outcomes Following Liver Retransplantation: Insights From a Retrospective Cohort Study

**DOI:** 10.3389/ti.2024.12104

**Published:** 2024-01-16

**Authors:** Felix J. Krendl, Margot Fodor, Madita L. Buch, Jessica Singh, Hannah Esser, Benno Cardini, Thomas Resch, Manuel Maglione, Christian Margreiter, Lisa Schlosser, Tobias Hell, Benedikt Schaefer, Heinz Zoller, Herbert Tilg, Stefan Schneeberger, Rupert Oberhuber

**Affiliations:** ^1^ Department of Visceral, Transplant and Thoracic Surgery, Center for Operative Medicine, Medical University of Innsbruck, Innsbruck, Austria; ^2^ Department of Mathematics, Innsbruck, Austria; ^3^ Department of Internal Medicine I, Gastroenterology, Hepatology, Endocrinology and Metabolism, Medical University of Innsbruck, Innsbruck, Austria

**Keywords:** risk factors, patient survival, graft survival, sepsis, futility

## Abstract

Liver retransplantation (reLT) yields poorer outcomes than primary liver transplantation, necessitating careful patient selection to avoid futile reLT. We conducted a retrospective analysis to assess reLT outcomes and identify associated risk factors. All adult patients who underwent a first reLT at the Medical University of Innsbruck from 2000 to 2021 (N = 111) were included. Graft- and patient survival were assessed via Kaplan-Meier plots and log-rank tests. Uni- and multivariate analyses were performed to identify independent predictors of graft loss. Five-year graft- and patient survival rates were 64.9% and 67.6%, respectively. The balance of risk (BAR) score was found to correlate with and be predictive of graft loss and patient death. The BAR score also predicted sepsis (AUC 0.676) and major complications (AUC 0.720). Multivariate Cox regression analysis identified sepsis [HR 5.179 (95% CI 2.575–10.417), *p* < 0.001] as the most significant independent risk factor for graft loss. At a cutoff of 18 points, the 5 year graft survival rate fell below 50%. The BAR score, a simple and easy to use score available at the time of organ acceptance, predicts and stratifies clinically relevant outcomes following reLT and may aid in clinical decision-making.

## Graphical Abstract

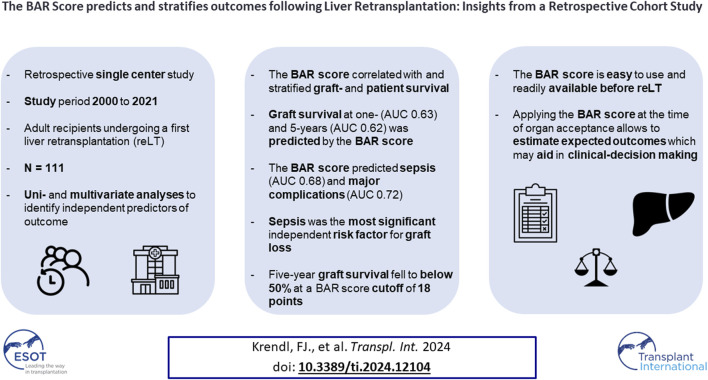

## Introduction

Liver transplantation (LT) is a curative treatment option for selected patients with end-stage liver disease and patients with certain forms of primary or secondary malignancies of the liver [1, 2]. In case of graft failure, a liver retransplantation (reLT) is the only recourse. Despite surgical, immunological and perioperative advancements, reLT remains a challenging procedure which is associated with inferior outcomes compared to primary LT [3–7].

The MELD score has been implemented by Eurotransplant and UNOS because it is an accurate predictor of short-term mortality and provides objective criteria for organ allocation in the majority of patients with end-stage liver disease [8, 9]. Patient selection for transplant recipients who require reLT is based on less well validated criteria and poses challenges in MELD-based allocation systems. Furthermore, outcomes following transplantation are not taken into consideration by current allocation policies [10].

In a field plagued by a shortage of available organs the need for reLT takes a toll on the already limited organ pool. Ethical principles such as utility, beneficence and equity need to be taken into consideration with patients on the waiting list competing for organs. It is therefore important to identify risk factors associated with negative outcomes in order to avoid futile retransplantations and maximize transplant benefit. While best achievable outcomes for LT and reLT have been quite well defined for selected standard risk (i.e., benchmark) cases [3, 11], futility and rationing remain concepts that are ill-defined [12, 13]. Previously, 5 year survival rates of 50% and more have been suggested to constitute an acceptable outcome [14]. Several risk scores have been published in an attempt to stratify risk and predict outcomes following reLT [15–18]. Yet, most of these scores are based on old data or lack adequate prediction of risk and are therefore of limited clinical applicability [18], which might explain why, so far, none of the published risk scores has found its way into routine clinical practice.

The aim of this study was to 1) evaluate the incidence of reLT in a high-volume Eurotransplant center, 2) assess graft- and patient survival as well as other relevant post-transplant complications following reLT and 3) identify potential risk factors associated with worse outcomes in the setting of reLT.

## Patients and Methods

### Study Population and Study Design

At the Medical University of Innsbruck, all adult patients who underwent a first deceased donor reLT between 1st January 2000 and 31st December 2021 were included in the study. Following discharge patients were routinely followed at our gastroenterology and hepatology outpatient clinic. Patient data were extracted from the electronic health records and pseudonymized. Data collection was performed from December 2022 until February 2023.

The study was conducted in accordance with both, the Declaration of Helsinki and Istanbul, and was approved by the Institutional Review Board of the Medical University of Innsbruck, Austria (EK1240/2022). The need for informed consent was waived by the ethics committee due to the retrospective nature of this study. The results were reported according to the STROBE guidelines [19].

### Surgical Technique

At our center, the standard implantation technique involves a bicaval, cava-replacing approach, without veno-venous bypass. Should individual circumstances preclude a cava-replacing approach, we employ a cava-sparing piggyback technique. Only if, 1) the hemodynamics of the patient preclude a cava-replacing approach and 2) the anatomical situation prevents a safe cava-sparing hepatectomy would we consider performing a bypass.

### Definitions

#### Graft Loss and Graft Dysfunction

Graft loss was defined as patient death or reLT (i.e., second reLT). Primary non-function (PNF) was defined as peak AST ≥3,000 IU/L plus at least one of the following criteria: INR ≥2.5, serum lactate ≥4 mmol/L and total bilirubin ≥10 mg/dL (values measured on postoperative day 3, biliary obstruction being excluded) [20]. Early allograft dysfunction (EAD) was defined according to the Olthoff criteria [21].

#### Rejections

Rejection episodes were diagnosed based on clinical suspicion and confirmed with liver biopsy. If rejection was suspected, patients received an intravenous steroid pulse of 500 mg methylprednisolone for 3 days followed by an increase in maintenance immunosuppression.

#### Infectious Complications and Sepsis

Any documented infection requiring some form of antimicrobial treatment was recorded as infectious complication. Sepsis was defined as a life-threatening organ dysfunction caused by a dysregulated host response to infection, in accordance with the third international consensus definitions for sepsis and septic shock [22].

#### Biliary Complications

Biliary complications were classified as bile duct leaks, anastomotic stenosis (AS), non-anastomotic stenosis (NAS) and cholangitis. Multifocal pathologies affecting the macroscopic donor bile ducts (NAS, biliary cast syndrome and bile duct necrosis with intrahepatic leakage and biloma formation) in the absence of thrombosis or severe stenosis of the hepatic artery that could not be explained by recurrent disease (i.e., primary sclerosing cholangitis) were classified as post-transplant cholangiopathy [23].

#### Balance of Risk (BAR) Score

The BAR score incorporates six variables (MELD score, donor age, recipient age, CIT, retransplantation and the need for life support) available at the time of organ acceptance and ranges from 0 to 27 points. BAR score values have been calculated according to the publication by Dutkowski et al. [24] using the online BAR score calculator.^1^

#### Classification and Quantification of Complications

Postoperative complications were graded according to the Clavien-Dindo classification system [25]. Clavien-Dindo grade I and II were recorded as minor complications. Clavien-Dindo Grade IIIa complications were considered moderate complications, while grade IIIb or higher were defined as major complications. Complications were further quantified using the comprehensive complication index (CCI) within a time frame of 3 months and 1 year after transplantation [26, 27].

### Outcomes

The primary outcomes were graft- and patient survival. Secondary outcomes included the incidence of post-transplant complications such as PNF, EAD, rejection episodes, infectious complications and sepsis, biliary and arterial complications as well as risk factors and their influence on graft loss and patient death.

### Statistical Analysis

For descriptive analyses, categorical variables were summarized with the help of absolute numbers and relative (percentages) frequencies, continuous variables were summarized with means and standard deviation (SD) or medians and interquartile range (IQR) as appropriate. Comparative analysis of categorical variables was conducted using the Chi-square or Fisher’s exact test (if one or more cells had an expected count of less than five). The Mann–Whitney U test was used to compare continuous, not normally distributed variables. Uni- and multivariate analyses were performed for the primary and secondary endpoints, starting with a univariate analysis of each variable. Any variable having a significant univariate test was selected as a candidate for the multivariate analysis [28]. Kaplan-Meier survival curves and the log-rank test were used to analyze and compare graft- and patient survival. Uni- and multivariate analysis for graft- and patient survival endpoints was performed with Cox proportional hazards regression analysis. Binary logistic regression analysis was used to assess the effects of clinical parameters on secondary outcomes. Potential associations between continuous variables were investigated with the help of bivariate correlation analysis using the Spearman correlation coefficient. Receiver operating characteristic (ROC) curves were plotted and areas under the curve (AUC) analyzed to evaluate the performance of binary classifiers. All *p*-values < 0.05 were considered statistically significant. Missing values were not imputed. Statistical analysis was conducted with SPSS (IBM SPSS Statistics for Mac, Version 27.0.1.0 Armonk, NY: IBM Corp.).

## Results

### Recipient Characteristics

Overall, 1,290 adult LTs were performed during the study period. Out of these 1,290 LTs, 111 (8.6%) were first reLTs. Indications for reLT and recipient demographics are presented in Table 1. The median recipient age was 57 years (50–65); 24 recipients (21.6%) were female, 87 were male (78.4%). The median recipient BMI was 23.5 (21.1–27.0). The most common indications for reLT were biliary complications (36.9%) followed by recurrence of disease (21.6%) and HAT (17.1%). The median time from primary LT to reLT was 13 months (2.0–66.0). Twenty-five patients (22.5%) underwent high urgency (HU) reLT. The median MELD score at reLT was 20 (14–26). The median BAR score in our cohort was 12 points (9–16) and ranged from 4 to 26 points. The median length of hospital stay was 32 days (20–55), with the median follow-up being 39.4 months (11.8–89.5).

**TABLE 1:** Recipient characteristics.

	All *N* = 111	Graft loss *n* = 52	Graft survival *n* = 59	*p*-value
Age (years)	57.0 (50.0–65.0)	56.0 (50.0–61.0)	59.0 (51.0–65.0)	0.11
Sex				0.08
- Female	24 (21.6)	15 (28.8)	9 (15.3)	
- Male	87 (78.4)	37 (71.2)	50 (84.7)	
BMI (kg/m^2^)	23.5 (20.8–27.0)	23.5 (20.7–26.8)	22.9 (21.1–27.0)	0.82
MELD score	20.0 (14.0–26.0)	21.50 (17.0–28.0)	17.0 (12.0–24.0)	**0.01**
BAR score	12.0 (9.0–16.0)	12.5 (11.0–16.0)	10.0 (8.0–16.0)	**0.04**
Indication for reLT				
- Biliary complications	41 (36.9)	21 (40.4)	20 (33.9)	0.48
- Disease recurrence	24 (21.6)	15 (28.8)	9 (15.3)	0.08
- HAT	19 (17.1)	6 (11.5)	13 (22.0)	0.14
- PNF	4 (3.6)	1 (1.9)	3 (5.1)	0.70
- Sepsis	1 (0.9)	1 (1.9)	0 (0.0)	0.95
- Rejection	9 (8.1)	2 (3.8)	7 (11.9)	0.23
- Other	10 (9.0)	4 (7.7)	6 (10.2)	0.90
- Not reported	3 (2.7)	2 (3.8)	1 (1.7)	0.91
Time to reLT (days)	406 (78–2010)	370 (79–2091)	406 (63–1,090)	0.79
AB induction (yes/no)	49 (45.0)	27 (54.0)	22 (37.3)	0.08
- IL2	44 (40.4)	24 (48.0)	20 (33.9)	0.14
- ATG	4 (3.7)	2 (4.0)	2 (3.4)	1.000
- Alemtuzumab	1 (0.9)	1 (2.0)	0 (0.0)	0.93
- Missing	2	0	2	
ABO blood group				
- A	50 (45.0)	21 (40.4)	29 (49.2)	0.35
- B	9 (8.1)	3 (5.8)	6 (10.2)	0.62
- 0	41 (36.9)	21 (40.4)	20 (33.9)	0.48
- AB	11 (9.9)	7 (13.5)	4 (6.8)	0.24
CMV mismatch				
- D+/R-	21 (19.6)	12 (24.0)	9 (15.8)	0.27
- D-/R+	39 (36.4)	17 (34.0)	22 (38.6)	0.62
- D+/R+	39 (36.4)	18 (36.0)	21 (36.8)	0.93
- D-/R-	8 (7.5)	3 (6.0)	5 (8.8)	0.86
- Missing	4	2	2	
Median follow-up (months)	39.4 (11.8–89.5)	6.0 (1.3–72.8)	67.0 (23.0–138.0)	

Values are presented as medians or absolute numbers with IQRs, and percentages in parentheses. Significant *p*-values are highlighted in bold. AB, antibody; ATG, anti-thymocyte globulin; BAR, balance of risk; BMI, body mass index; CMV, cytomegalovirus; MELD, model for end-stage liver disease; HAT, hepatic artery thrombosis; IL2, interleukin 2; IQR, interquartile range; PNF, primary non-function; reLT, liver retransplantation.

### Donor Characteristics and Operative Factors

The median donor age was 46 years (32–54); 55 donors (49.5%) were female, 56 (50.5%) were male (Table 2). The median ET-DRI was 1.44 (1.25–1.73), with the median donor BMI being 24.8 (23.0–27.0). All donors were donation after brain death (DBD) donors. The anhepatic time, warm ischemia time (WIT) and cold ischemia time (CIT) were 57.0 min (48.0–66.0), 45.0 min (37.0–56.0) and 8.1 h (6.5–9.5) respectively. The median operating time was 7.6 h (6.0–8.9). Cava-replacing LT was performed in 93.7% (104 of 111) of cases with piggy-back transplantation being performed in 6.3% of cases (7 of 111). An arterial jump graft was used in 24.3% of cases (27 of 111) and a bilioenteric anastomosis was carried out in 36.0% of cases (40 of 111). In 14.4% of cases (16 of 111) the liver graft had undergone normothermic machine perfusion before implantation.

**TABLE 2:** Donor characteristics and operative data.

	All *N* = 111	Graft loss *n* = 52	Graft survival *n* = 59	*p*-value
Age (years)	46.0 (32.0–54.0)	49.0 (34.0–54.8)	42.0 (30.0–54.0)	0.16
Sex				0.07
- Female	55 (49.6)	21 (40.4)	34 (57.6)	
- Male	56 (50.5)	31 (59.6)	25 (42.4)	
BMI (kg/m^2^)	24.8 (22.9–29.0)	25.4 (23.0–28.0)	24.2 (23.0–26.0)	0.19
COD				0.58
- Trauma	35 (31.5)	17 (32.7)	18 (30.5)	
- Anoxia	7 (6.3)	4 (7.7)	3 (5.1)	
- CVA	65 (58.6)	30 (59.3)	35 (57.7)	
- Other	4 (3.6)	1 (1.9)	3 (5.1)	
ECD	74 (66.7)	32 (61.5)	37 (62.7)	0.90
DCD	0 (0.0)	0 (0.0)	0 (0.0)	
DBD	111 (100.0)	52 (100.0)	59 (100.0)	
NMP	16 (14.4)	4 (7.7)	12 (20.3)	0.06
Preservation				0.64
- UW	37 (33.6)	16 (31.4)	21 (35.6)	
- HTK	73 (66.4)	35 (68.6)	38 (64.4)	
- Missing	1	1	0	
reLT era				0.06
- 2000–2010	41 (36.9)	24 (46.2)	17 (28.8)	
- 2011–2021	70 (63.1)	28 (53.8)	42 (71.2)	
Duration reLT (hours)	7.6 (6.0–8.9)	7.6 (5.9–9.4)	7.6 (6.3–8.7)	0.83
Anhepatic time (minutes)	57.0 (48.0–66.0)	56.0 (45.3–70.0)	57.0 (50.0–66.0)	0.79
WIT (minutes)	45.0 (37.0–56.0)	45.0 (37.0–55.0)	44.0 (37.8–58.0)	0.84
CIT (hours)	8.1 (6.5–9.5)	8.7 (6.8–10.6)	7.7 (6.3–9.3)	**0.02**
ET-DRI	1.44 (1.25–1.73)	1.48 (1.34–1.71)	1.41 (1.13–1.82)	0.34

Values are presented as medians or absolute numbers with IQRs, and percentages in parentheses. Significant *p*-values are highlighted in bold. BMI, body mass index; COD, cause of death; CVA, cerebrovascular accident; ECD, extended criteria donor; ET-DRI, Eurotransplant donor risk index; HTK, histidine-tryptophan-ketoglutarate. IQR, interquartile range; SAB, subarachnoid hemorrhage; UW, University of Wisconsin; WIT, warm ischemia time.

### Complications Following reLT

Six patients (5.4%) developed primary non-function (PNF) following reLT. The EAD rate was 35.1% (39 of 111). Ten recipients (9.0%) had a rejection episode; 78 patients (70.3%) developed infectious complications. Sepsis occurred in 22 patients (19.8%). Overall, 11 patients (9.9%) developed an arterial complication; HAT occurred in four patients (3.6%) with arterial stenosis (*n* = 4, 3.6%), dissection (*n* = 1, 0.9%) and pseudoaneurysm (*n* = 2, 1.8%) being responsible for the other arterial complications.

Out of 111 patients, 54 (48.6%) developed a biliary complication. Seventeen patients (15.3%) had one or more cholangitis episode. Bile duct leaks occurred in 19.8% (22 of 111), anastomotic strictures in 24.3% (27 of 111), non-anastomotic strictures in 10.8% (12 of 111) and post-transplant cholangiopathy in 17.1% (19 of 111) of the recipients (Table 3). Patients with biliary complications tended to have a higher graft loss rate compared to patients without biliary complications, however the difference was not statistically significant [27.0% (30 of 111) vs. 20.7% (23 of 111), *p* = 0.11].

**TABLE 3:** Clinical outcomes and complications.

	All *N* = 111	Graft loss *n* = 52	Graft survival *n* = 59	*p*-value
PNF	6 (5.4)	6 (11.5)	0 (0.0)	**0.02**
EAD	39 (35.5)	20 (39.2)	19 (32.2)	0.44
Rejection	10 (9.1)	7 (13.5)	3 (5.1)	0.23
Infectious complications	78 (72.2)	38 (74.5)	40 (69.0)	0.52
Sepsis	22 (19.8)	19 (38.0)	3 (5.1)	**<0.001**
Biliary complications	54 (48.6)	30 (57.7)	24 (40.7)	0.07
- Cholangitis	17 (15.3)	12 (23.1)	5 (8.5)	**0.03**
- Bile duct leaks	22 (19.8)	12 (23.1)	10 (16.9)	0.42
- AS	27 (24.3)	17 (32.7)	10 (16.9)	0.05
- NAS	12 (10.8)	8 (15.4)	4 (6.8)	0.15
- Post-Tx Cholangiopathy	19 (17.1)	11 (21.2)	8 (13.6)	0.29
Arterial complications	11 (9.9)	8 (15.4)	3 (5.1)	0.07
- Stenosis	4 (3.6)	3 (5.8)	1 (1.7)	0.52
- Thrombosis	4 (3.6)	3 (5.8)	1 (1.7)	0.52
- Dissection	1 (0.9)	1 (1.9)	0 (0.0)	0.95
- Pseudoaneurysm	2 (1.8)	1 (1.9)	1 (1.7)	1.000
Major complication (at discharge)	91 (82)	47 (92.2)	44 (77.2)	**0.03**
CCI				
- 3 months	54.2 (39.7–86.5)	68.1 (51.0–100)	47.3 (26.2–69.0)	**<0.001**
- 12 months	63.9 (42.4–100)	100 (63.8–100)	54.2 (33.7–75.7)	**<0.001**
Reoperation	69 (62.2)	37 (71.2)	32 (54.2)	0.07
- Reoperation ≤30 days	64 (57.7)	35 (67.3)	29 (49.2)	0.05
Hospital stay (days)	32 (20–55)	38 (20–59)	29 (22–46)	0.55

Values are presented as medians or absolute numbers with IQRs, and percentages in parentheses. Significant *p*-values are highlighted in bold. AS, anastomotic stricture; CCI, comprehensive complication index; EAD, early allograft dysfunction; NAS, non-anastomotic stricture; PNF, primary non function; Post-Tx, post-transplant.

### Graft Survival Analysis

**FIGURE 1:**
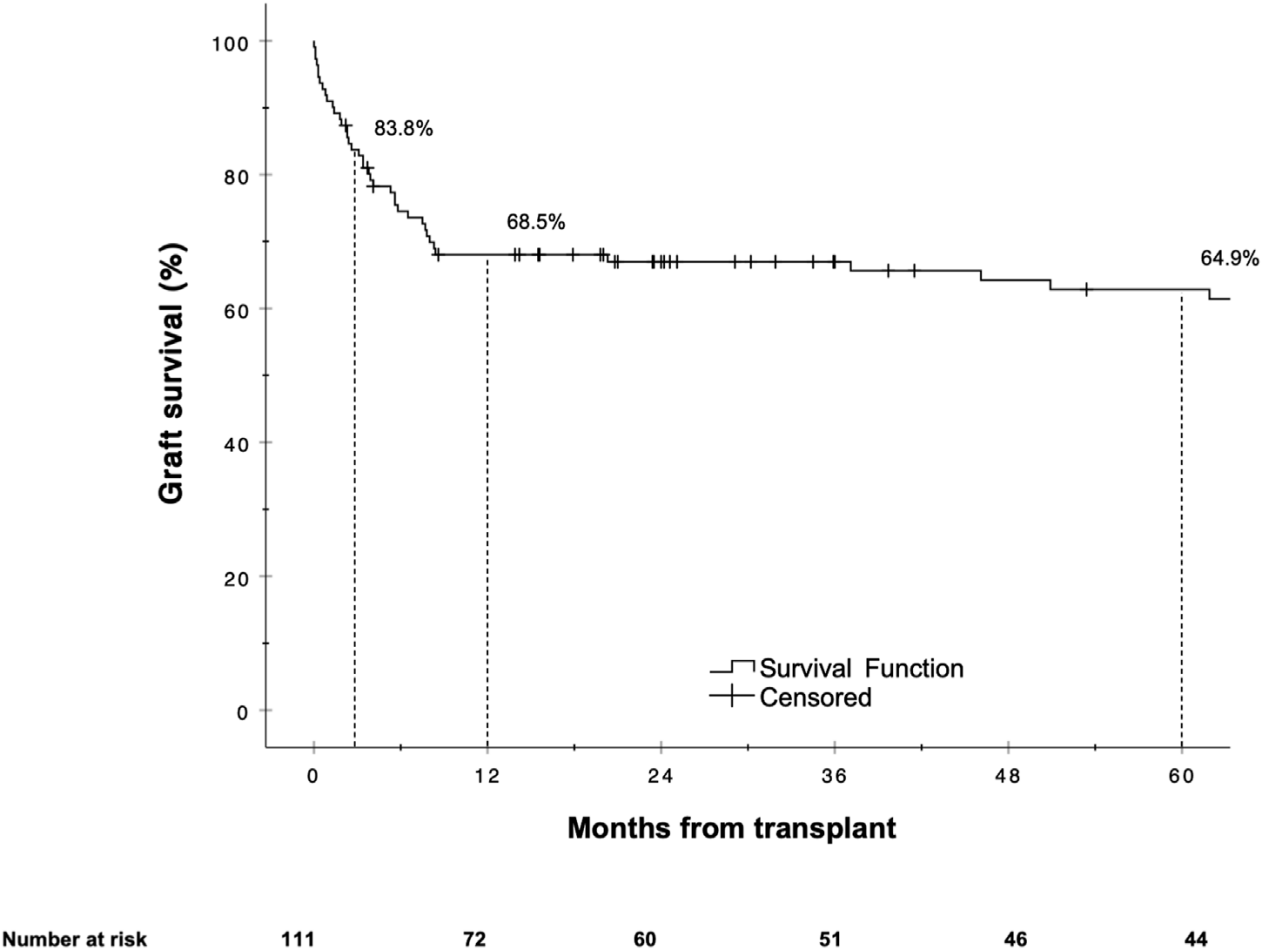
Estimated Kaplan-Meier graft survival for the reLT cohort.

**FIGURE 2:**
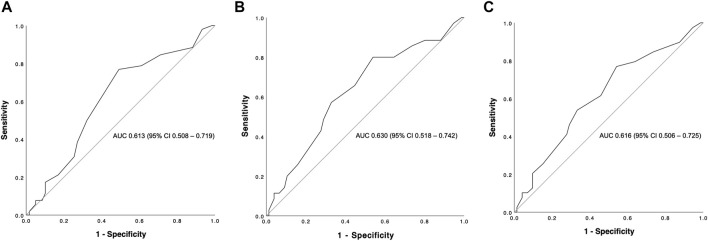
ROC curve analysis depicting the predictive capability of the BAR score for overall **(A)**, 1 **(B)** and 5 year **(C)** graft survival. The BAR score performed best at predicting 1 year graft survival (AUC 0.630).

The overall graft failure rate (patient death or reLT) was 46.8% (52 of 111) over the observation period of 22 years. Out of these 52 patients, seven underwent a second reLT and 45 died with their second graft. The most common cause of graft failure was sepsis (34.6%) followed by recurrence of disease (17.3%), vascular complications (15.4%) and post-transplant malignancies (9.6%). Kaplan Meier estimates for 90 days, 1 and 5 year graft survival are shown in Figure 1. Graft survival was significantly associated with the BAR score in univariate analysis. ROC curve analysis showed the BAR score to be predictive of overall [AUC 0.613 (95% CI 0.508–0.719), *p* = 0.04], 1 [AUC 0.630 (95% CI 0.518–0.742), *p* = 0.03] and 5 year graft loss [AUC 0.616 (95% CI 0.506–0.725), *p* = 0.045] but not 90 days graft loss [AUC 0.640 (95% CI 0.477–0.803), *p* = 0.06] (Figure 2).

Different BAR score values were analyzed to find optimal cutoffs which best stratify risk of graft failure at different time points following reLT. Cutoffs were based on the maximum Youden-index [29].

**FIGURE 3:**
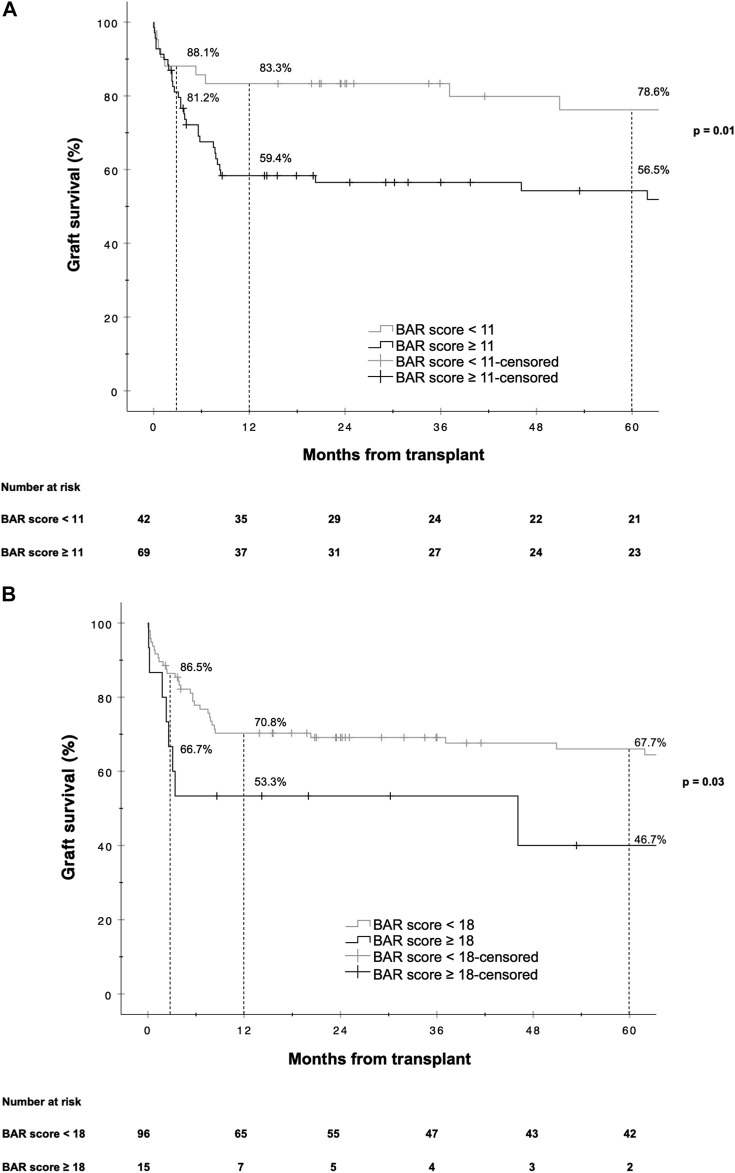
Kaplan-Meier survival curves showing graft survival for reLT recipients with a BAR score <11 points vs. ≥11 points **(A)** and <18 points vs. ≥18 points **(B)**. The difference in graft survival at 1 and 5 years following reLT was highest at a BAR score cutoff of 11 points. At a BAR score cutoff of 18 points 5 year graft survival fell to below 46.7%.

A BAR score cutoff of 11 points (BAR score <11 points vs. ≥11 points) provided the best separation of risk. At this cutoff the positive predictive value (PPV) for graft failure at 1 and 5 years was 40.6% and 43.5% respectively, while the negative predictive value (NPV) was 83.3% and 78.6% respectively. Patients with a BAR score ≥11 points had an increased hazard of graft loss at 1 [HR 2.784 (95% CI 1.215–6.381), *p* = 0.02] and 5 years [HR 2.396 (95% CI 1.136–5.055), *p* = 0.02] compared to patients with a BAR score <11 points. At a cutoff of 18 points the 5 year graft survival rate fell to 46.7% (Figure 3).

Univariate analysis revealed MELD score, donor age, CIT, BAR score, cholangitis, major complication (CD > IIIa), sepsis, reoperation within 30 days and PNF to be risk factors for graft loss.

Considering these factors for multivariate Cox regression analysis, donor age, PNF and sepsis remained as independent risk factors for graft loss (Supplementary Table S1). When excluding the MELD score, donor age and CIT (all parameters are included in the BAR score) as well as PNF (PNF invariably leading to graft loss, Table 3) from the multivariate Cox regression, only sepsis [HR 5.179 (95% CI 2.575–10.417, *p* < 0.001] remained as independent significant risk factor for graft loss (Table 4).

**TABLE 4:** Graft survival–Multivariate adjusted Cox proportional hazards regression analysis^a^.

	HR	95% CI	*p*-value
BAR score	1.019	0.952–1.091	0.59
Cholangitis	1.934	0.959–3.901	0.07
Major complication	1.490	0.453–4.907	0.51
Sepsis	5.179	2.575–10.417	**<0.001**
Reoperation within 30 days	1.279	0.619–2.640	0.51

BAR, balance of risk; CI, confidence interval; HR, hazard ratio.

^a^ The MELD score, donor age and CIT (all included in the BAR score) as well as PNF have been excluded.

### Patient Survival Analysis

The overall mortality rate was 45% (50 of 111). The in-hospital mortality rate was 16.2% (18 of 111).

**FIGURE 4:**
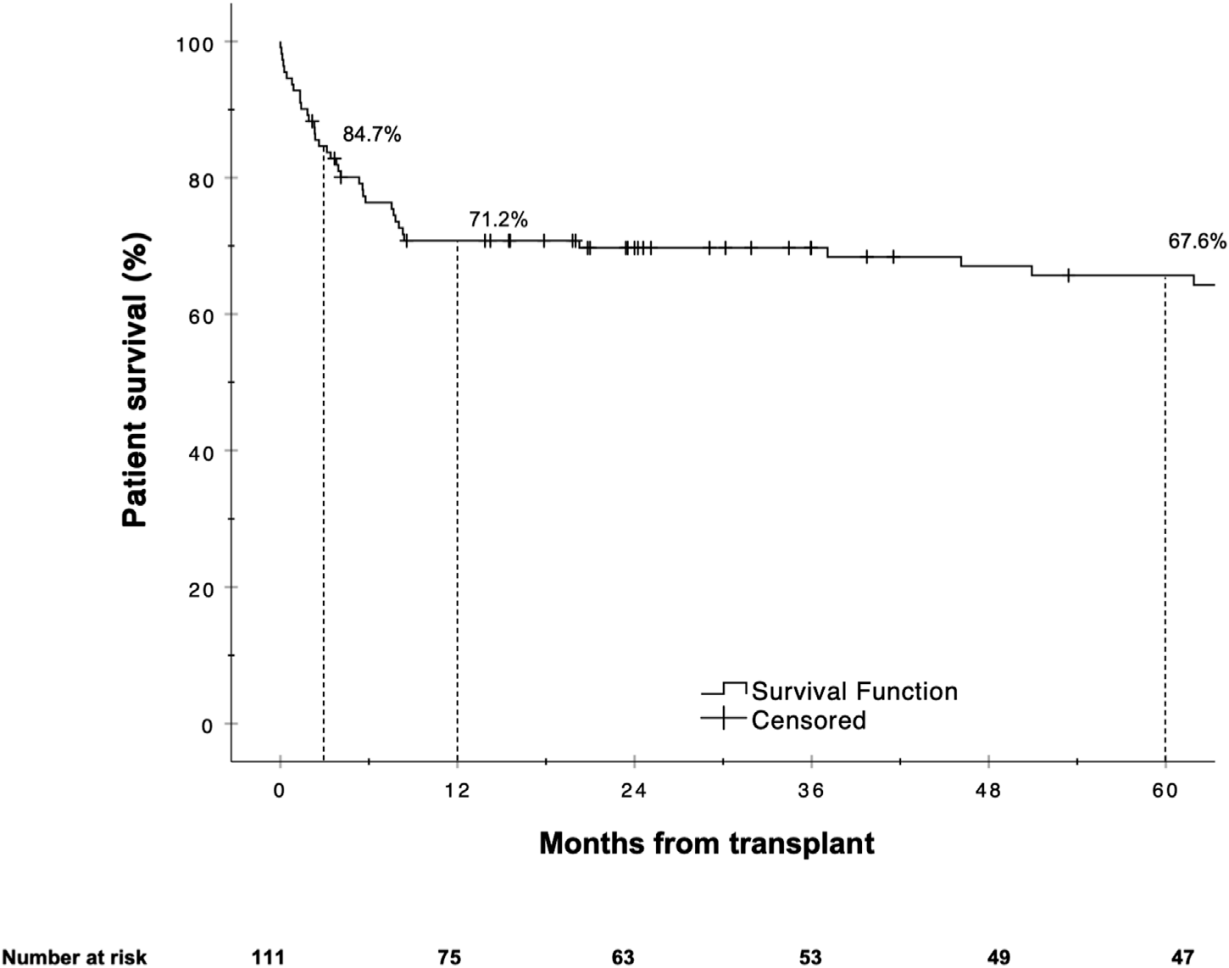
Estimated Kaplan-Meier patient survival for the reLT cohort.

**FIGURE 5:**
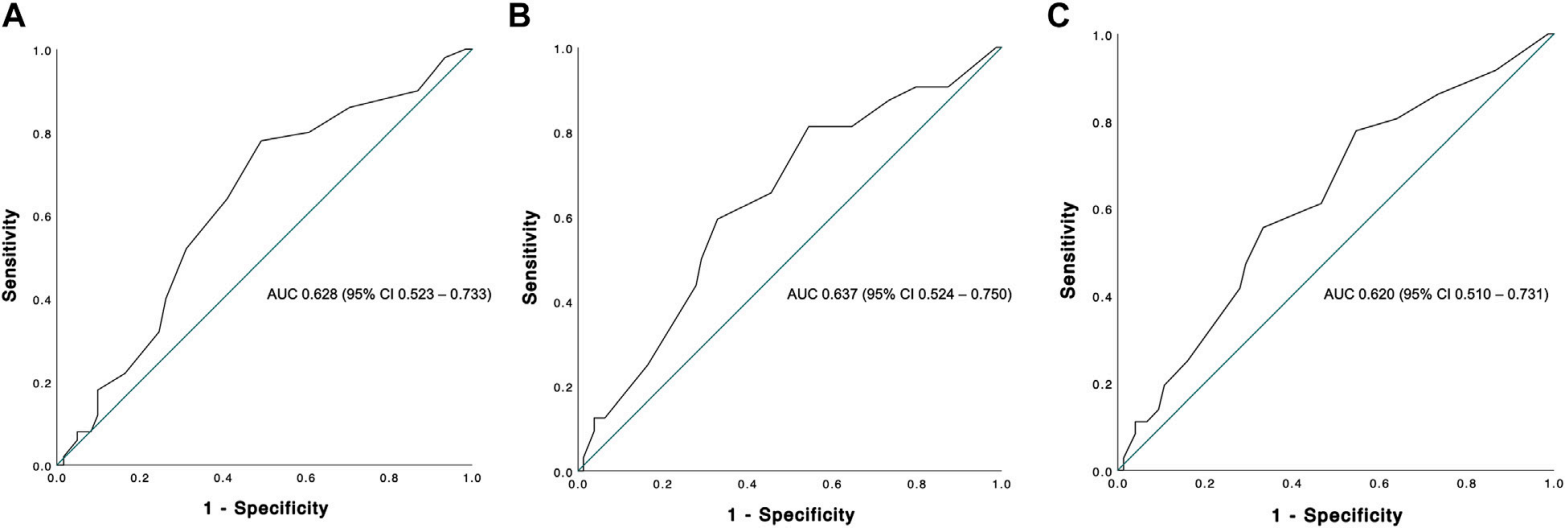
ROC curve analysis depicting the predictive capability of the BAR score for overall **(A)**, 1 **(B)** and 5 years **(C)** patient survival. The BAR score performed best at predicting 1 year patient survival (AUC 0.637).

The Kaplan-Meier estimates for 90 days, 1 and 5 year patient survival are shown in Figure 4. Similar to graft survival, patient survival was significantly associated with the BAR score in univariate analysis. ROC curve analysis showed the BAR score to be predictive of overall [AUC 0.628 (95% CI 0.523–0.733), *p* = 0.02], 1 [AUC 0.637 (95% CI 0.524–0.750), *p* = 0.02] and 5 year patient death [AUC 0.620 (95% CI 0.510–0.731), *p* = 0.04] but not 90 days mortality [AUC 0.644 (95% CI 0.473–0.816), *p* = 0.06] (Figure 5).

The BAR score cutoff with the best separation of risk for patient death was the same as for graft survival (11 points). The PPV for patient death at 1 and 5 years was 37.7% and 40.6% respectively. The NPV at 1 and 5 years was 85.7% and 81.0% respectively.

**FIGURE 6:**
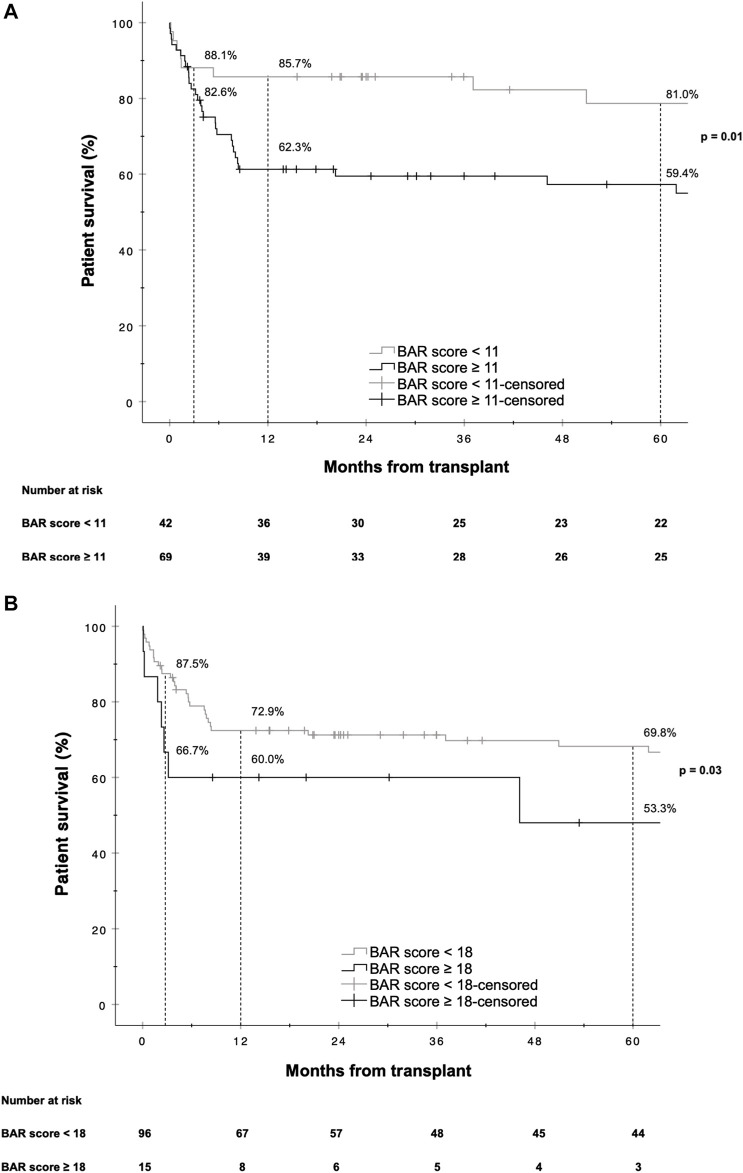
Kaplan-Meier survival curves showing patient survival for reLT recipients with a BAR score <11 points vs. ≥11 points **(A)** and <18 points vs. ≥18 points **(B)**. The difference in graft survival at 1 and 5 years following reLT was highest at a BAR score cutoff of 11 points. At a BAR score cutoff of 18 points 5 year graft survival fell to below 53.3%.

The hazard ratios for 1 and 5 year mortality at a BAR score cutoff of 11 were [HR 2.963 (95% CI 1.218–7.205), *p* = 0.02] and [HR 2.474 (95% CI 1.126–5.435), *p* = 0.02] respectively. At a BAR score cutoff of 18 points 5 year patient survival dropped to 53.3% (Figure 6).

Univariate analysis revealed the MELD score, donor age, BAR score, cholangitis, major complications (CD > IIIa), PNF, sepsis, arterial complications, and reoperation within 30 days as risk factors for patient death. Considering these factors for multivariate Cox regression analysis the most significant independent risk factors for patient death were PNF, sepsis and donor age (Supplementary Table S2). In a separate model where the MELD score and donor age (both included in the BAR score) have been excluded from the multivariate Cox regression analysis, PNF [HR 29.987 (95% CI 7.514–119.664), *p* < 0.001], and sepsis [HR 3.755 (95% CI 1.819–7.751), *p* < 0.001] remained as the only independent risk factors for patient death (Table 5).

**TABLE 5:** Patient survival–multivariate adjusted Cox proportional hazards regression analysis^a^.

	HR	95% CI	*p*-value
BAR score	1.050	0.981–1.125	0.16
Cholangitis	1.558	0.729–3.328	0.25
Major complication	1.427	0.430–4.737	0.56
PNF	29.987	7.514–119.664	**<0.001**
Sepsis	3.755	1.819–7.751	**<0.001**
Arterial complication	1.601	0.656–3.907	0.30
Reoperation within 30 days	1.002	0.473–2.120	0.996

BAR, balance of risk; CI, confidence interval; HR, hazard ratio; PNF, primary non-function.

^a^ The MELD, score and donor age (both included in the BAR, score) have been excluded.

### BAR Score

In our analysis, the BAR score not only correlated significantly with graft- and patient survival but also with sepsis [OR 1.146 (CI 95% 1.035–1.269), *p* = 0.01], major complications (CD > IIIa) at discharge [OR 1.236 (CI 95% 1.064–1.437), *p* = 0.01] and the duration of the hospital stay (Spearman’s r = 0.329, *p* < 0.001) as well as CCI at 3 (Spearman’s r = 0.318, *p* < 0.001) and 12 months (Spearman’s r = 0.272, *p* = 0.004). The BAR score was highly predictive of the incidence of major complications (CD > IIIa) with an AUC of 0.720 (95% CI 0.613–0.828, *p* = 0.004) and the occurrence of sepsis [AUC 0.676 (CI 95% 0.548–0.804), *p* = 0.01]. The BAR score correlated with the ET-DRI (r = 0.213, *p* < 0.02) (the scores share two variables: donor age and CIT). However, in comparison to the BAR score the ET-DRI was not predictive of patient- [AUC 0.522 (CI 95% 0.405–0.639), *p* = 0.72] or graft survival [AUC 0.568 (CI 95% 0.461–0.676), *p* = 0.21].

In response to the strong correlation of the BAR score with major complications and sepsis we performed additional Cox regression analysis excluding these two parameters from the multivariate model to avoid any interference, after which the BAR score remained as a significant independent risk factor for graft loss and patient death (Supplementary Tables S3, S4).

### Sepsis and PNF

In our analysis, sepsis and PNF were the strongest independent predictors of graft failure and patient death in the multivariate Cox regression models. Univariate binary logistic regression analysis revealed EAD [OR 2.769 (CI 95% 1.063–7.211), *p* = 0.04], reoperation within 30 days [OR 3.030 (CI 95% 1.027–8.945), *p* = 0.045], BAR score [OR 1.146 (CI 95% 1.035–1.269), *p* = 0.01] and MELD score [OR 1.092 (CI 95% 1.029–1.159), *p* = 0.003] to correlate with sepsis. Considering EAD, reoperation within 30 days and the BAR score for multivariate binary logistic regression analysis only the BAR score remained significantly associated with sepsis [OR 1.122 (CI 95% 1.011–1.254), *p* = 0.03]. No single parameter was found to correlate with PNF.

## Discussion

This study, evaluating outcomes following reLT over the course of a 22 years period, found the BAR score to correlate with and be predictive of graft loss and patient death as well as the occurrence of sepsis and major complications. Furthermore, the BAR score positively correlated with the CCI at 3 and 12 months as well as the duration of hospital stay. The incidence of reLT over the duration of the study period was 8.6% and is in line with those reported at other transplant centers [6].

Multivariate Cox regression analysis revealed sepsis and PNF to be the strongest independent risk factors of graft failure and patient death. The overall morbidity and mortality were high with more than 80% of recipients developing a major complication and a 5 year patient survival below 70%, underscoring the high-risks associated with reLT.

Previously, an expected 5 year patient survival of 50% or more has been demanded to justify LT [30, 31].

Schlegel et al. have defined futility as in-hospital or 90 days mortality [32]. At a recent consensus meeting the expert panel recommended patient- and graft survival at 1 year after LT to define futility [33]. In the context of reLT an expected 1 year patient survival of 50% and more as well as an expected 5 year graft survival above 50% have been suggested as minimum thresholds to define acceptable outcomes [14]. While this is an arbitrary cutoff it also lacks clinical feasibility since outcome projections in reLT are ill-defined.

In our cohort, the hazard of graft loss and patient death was highest in the first months following reLT with survival curves running almost parallel after the first year (Figures 3, 6). In line with this observation, the BAR score performed best at predicting risk of patient death at 1 year (AUC 0.637) and 1 year graft loss (AUC 0.630) (Figures 2, 5).

The AUCs reported for the BAR score in our cohort were higher compared to those of previously published risk models for reLT and similar to the AUC for the model published in 2011 by Hong et al. (AUC 0.64) as well as the recently published Liver Retransplant Risk Score by Brüggenwirth et al. (time-dependent AUC for graft loss at 1 year 0.623) [17, 18]. The Liver Retransplant Risk Score was developed from a large dataset of the European Liver Transplant Registry (ELTR) and externally validated, however levels of pretransplant bilirubin, creatinine and INR were missing in more than 50% of cases with MELD score and CIT missing in 48% and 38% of cases respectively.

The Liver Retransplant Risk Score uses similar parameters as the BAR score (donor and recipient age, CIT, MELD score, life support before reLT), substituting the need for life support prior to reLT with hospitalization before reLT, and adds two new variables: indication for reLT and time to reLT. Both factors were analyzed in the present study but not significantly associated with neither graft- nor patient survival (Supplementary Tables S5, S6). Consistent with our observations, other authors also found the indication for reLT as well as the time interval from LT to reLT not to be associated with graft- or patient survival [4, 34].

Similar to our observation, Brüggenwirth et al. discovered that the discriminating power of the Liver Retransplant Risk Score is most prominent in the first 6 to 12 months following reLT with survival curves running parallel thereafter [18]. Correspondingly, Yoon et al. also observed the most significant decline in survival during the first year following reLT [4].

In addition to its predictive value for graft- and patient survival, we observed the BAR score to exhibit a moderate positive correlation with the duration of hospital stay (r = 0.329, *p* < 0.001) and the CCI at 3 and 12 months (r = 0.272, *p* = 0.004). Furthermore, the BAR score showed good prediction of the incidence of major complications (AUC 0.720, *p* = 0.004). These findings are consistent with those previously reported by Schlegel et al. and Boecker et al. [32, 35]. In our cohort, the BAR score also predicted the incidence of sepsis–among the strongest independent risk factors of graft loss and patient death–with reasonable accuracy (AUC 0.676, *p* = 0.01).

An ideal predictive score is simple and easy to use, incorporates relevant donor and recipient factors and is available at the time of organ acceptance. The BAR score fulfils all these criteria. Among risk scores which are based on data available at the time of organ acceptance, the BAR score performs best and its robustness in predicting post-transplant outcomes in various settings has been shown in multiple studies including ours [36–38].

Various BAR score cutoffs have been suggested in the past. Boecker et al. found a cutoff of 14 points to best predict risk when analyzing 90 days patient- and graft survival following LT. However, 5 year patient survival was only moderately stratified at this cutoff (76% vs. 69%) [35].

Martínez et al. reported a cutoff of 15 points to best discriminate risk of 3 months, 1 and 5 year mortality [39], while Zakareya et al. determined that a cutoff of 10 points is best at predicting risk of patient death at 3 months, 1 and 5 years [40]. In our analysis a BAR score cutoff of 11 points exhibited the highest discriminating power in terms of graft loss and patient death at 1 and 5 years. Dutkowski et al. proposed a BAR score cutoff of 18 points as they observed that 5 year survival rates start to decline exponentially beyond this point [24]. In line with this observation, we found that 5 year graft survival dropped to below 50% for recipients with a BAR score ≥18 points (Figure 3).

With different definitions of futility and rationing in use, the transplant community often refers to a 5 year survival rate of 50% or higher as the threshold for an acceptable outcome [14]. However, futility and rationing will mean different things to different people in different contexts. The local waitlist dynamics as well as the availability of a potential live donor program will certainly impact the decision-making process. Given these complexities, it is difficult to recommend a definitive BAR score cutoff although a score around 18 points seems to mark a transition zone where outcomes are declining below what is considered acceptable. Consequently, optimizing donor-recipient combinations needs to be at the forefront of medical decision-making when accepting organs. In our study, good quality grafts–signified by the low median ET-DRI (1.44)–were used.

In the end, maximizing transplant benefit (i.e., the life years gained with LT as opposed to remaining on the waiting list) for the individual patient must be the main goal. To achieve this goal, transplant programs must be conscientious of their local circumstances including waitlist dynamics, recipient risk profiles and organ availability.

### Limitations and Strengths

The present study has several limitations, which are mostly related to its retrospective study design. Although the overall number of LTs performed at the Medical University of Innsbruck was quite high, the sample size was limited since the proportion of reLTs was below 10%. The small sample size may have limited the statistical power especially for outcomes with low event rates. Furthermore, even though the BAR score has been shown to have the best predictive capability of all risk scores which are based on data available at the time of organ acceptance, better scores with AUCs well above 0.7 for relevant clinical outcome measures would be desirable.

Strengths of our study include the prospectively maintained LT database at our center and the high data granularity with little to no missing data in comparison to large registry studies. Moreover, the clinical management—from recipient evaluation to donor organ selection, surgical procedures and post-transplant care–was fairly homogenous at our center despite the long observation period. Still, multi-center studies evaluating clinical risk scores in the setting of reLT are needed.

## Conclusion

The BAR score, a simple and readily available score available at the time of organ acceptance, is predictive of graft- and patient survival as well as duration of hospital stay, occurrence of sepsis and major complications following reLT. A cutoff of 11 points demonstrated the best discriminating power in terms of graft loss and patient death (i.e., the difference in survival between groups was highest at this cutoff). The hazards of graft loss and patient death were highest in the first year following reLT. In line with this observation, the BAR score performed best at predicting the 1 year risk of graft loss and patient death, comparing favorably to previously published reLT risk scores. For recipients with a BAR score ≥18 points, 5 year patient- and graft survival rates dropped to 50% and below. Sepsis and PNF were the strongest independent risk factors of graft loss and patient death. The occurrence of sepsis was predicted by the BAR score. In summary, the BAR score may serve as a predictive tool, allowing clinicians to estimate expected outcomes thereby facilitating clinical decision-making.

## Figures and Tables

**Table:** 

**AUC**	area under the curve
**Aza**	azathioprine
**AS**	anastomotic stenosis
**BAR**	balance of risk
**BMI**	body mass index
**CCI**	comprehensive complication index
**CIT**	cold ischemia time
**CsA**	cyclosporine a
**DBD**	donation after brain death
**DCD**	donation after cardiocirculatory death
**EAD**	early allograft dysfunction
**ECD**	extended criteria donor
**EK**	ethikkommission (institutional review board)
**ET-DRI**	eurotransplant donor risk index
**HAT**	hepatic artery thrombosis
**HR**	hazard ratio
**HTK**	histidine-tryptophan-ketoglutarate
**IL2**	interleukin 2
**INR**	international normalized ratio
**IQR**	interquartile range
**LT**	liver transplantation
**MELD**	model for end-stage liver disease
**MMF**	mycophenolate mofetil
**MPA**	mycophenolic acid
**PNF**	primary non-function
**reLT**	liver retransplantation
**SD**	standard deviation
**STROBE**	strengthening the reporting of observational studies in epidemiology
**Tac**	tacrolimus
**UNOS**	united network for organ sharing
**UW**	university of wisconsin
**WIT**	warm ischemia time

## Data Availability

Data are available from the corresponding author upon reasonable request. Requests to access these datasets should be directed to rupert.oberhuber@i-med.ac.at.

## Footnotes

1
https://www.assessurgery.com/bar-score/bar-score-calculator/
