# Supplementary material for: The BAR Score Predicts and Stratifies Outcomes Following Liver Retransplantation: Insights From a Retrospective Cohort Study
Source: Transpl Int. 2024 Jan 16;37:12104. doi: 10.3389/ti.2024.12104 (PMC10833230; doi:10.3389/ti.2024.12104)
Supplement: Supplementary file 1 [file DataSheet1.docx]

**Supplementary Digital Content**

| **Table S1. Graft survival – Multivariate cox proportional hazard regression analysis.** | | | |
| --- | --- | --- | --- |
|  | **HR** | **95 % CI** | **P-value** |
| **MELD score** | 1.066 | 0.994 – 1.144 | 0.07 |
| **Donor Age** | 1.029 | 1.008 – 1.052 | **0.01** |
| **CIT** | 1.001 | 0.999 – 1.003 | 0.26 |
| **BAR score** | 0.935 | 0.826 – 1.058 | 0.29 |
| **Cholangitis** | 1.872 | 0.911 – 3.847 | 0.09 |
| **Major complications** | 1.279 | 0.382 – 4.283 | 0.69 |
| **Sepsis** | 5.047 | 2.427 – 10.495 | **< 0.001** |
| **Reoperation within 30 days** | 1.033 | 0.482 – 2.211 | 0.93 |
| **PNF** | 33.453 | 8.468 – 132.154 | **< 0.001** |
| BAR, balance of risk; CI, confidence interval; CIT, cold ischemia time; HR, hazard ratio; MELD, model of end-stage disease; PNF, primary non-function. | | | |

| **Table S2. Patient survival – Multivariate cox proportional hazard regression analysis.** | | | |
| --- | --- | --- | --- |
|  | **HR** | **95 % CI** | **P-value** |
| **MELD score** | 1.066 | 0.993 – 1.145 | 0.078 |
| **Donor age** | 1.026 | 1.004 – 1.048 | **0.02** |
| **BAR score** | 0.969 | 0.859 – 1.092 | 0.60 |
| **Cholangitis** | 1.662 | 0.780 – 3.541 | 0.19 |
| **Major complication** | 1.206 | 0.359 – 4.055 | 0.76 |
| **PNF** | 32.449 | 7.577 – 138.958 | **< 0.001** |
| **Sepsis** | 3.994 | 1.929 – 8.269 | **< 0.001** |
| **Arterial complication** | 1.298 | 0.510 – 3.307 | 0.58 |
| **Reoperation within 30 days** | 0.939 | 0.432 – 2.042 | 0.88 |
| BAR, balance of risk; CI, confidence interval; HR, hazard ratio; MELD, model of end-stage disease; PNF, primary non-function. | | | |

| **Table S3. Graft survival – Multivariate cox proportional hazard regression analysis.*** | | | |
| --- | --- | --- | --- |
|  | **HR** | **95 % CI** | **P-value** |
| **BAR score** | 1.075 | 1.012 – 1.142 | **0.02** |
| **Cholangitis** | 2.163 | 1.097 – 4.263 | **0.03** |
| **Reoperation within 30 days** | 1.844 | 1.022 – 3.325 | **0.04** |
| BAR, balance of risk; CI, confidence interval; HR, hazards ratio; PNF, primary non-function. * Major complications, sepsis and PNF excluded. | | | |

| **Table S4. Patient survival – Multivariate cox proportional hazard regression analysis.*** | | | |
| --- | --- | --- | --- |
|  | **HR** | **95 % CI** | **P-value** |
| **BAR score** | 1.093 | 1.026 – 1.163 | **0.01** |
| **Cholangitis** | 2.071 | 1.015 – 4.226 | **0.05** |
| **PNF** | 26.713 | 8.326 – 85.705 | **< 0.001** |
| **Arterial complication** | 1.571 | 0.682 – 3.617 | 0.29 |
| **Reoperation within 30 days** | 1.385 | 0.747 – 2.565 | 0.30 |
| BAR, balance of risk. CI, confidence interval. HR, hazards ratio; PNF, primary non-function. *Major complications and sepsis excluded. | | | |

| **Table S5. Patient survival – Univariate cox proportional hazard regression analysis.*** | | | |
| --- | --- | --- | --- |
|  | **HR** | **95 % CI** | **P-value** |
| **Time to reLT** | 1.000 | 1.000 – 1.000 | **0.23** |
| - 2 weeks – 3 months | 1.697 | 0.841 – 3.424 | **0.14** |
| - Within 30 days | 0.721 | 0.323 – 1.607 | **0.72** |
| CI, confidence interval. HR, hazards ratio; PNF, primary non-function. *Time to reLT. | | | |

| **Table S6. Graft survival – Univariate cox proportional hazard regression analysis.*** | | | |
| --- | --- | --- | --- |
|  | **HR** | **95 % CI** | **P-value** |
| **Time to reLT** | 1.000 | 1.000 – 1.000 | **0.07** |
| - 2 weeks – 3 months | 1.531 | 0.762 – 3.076 | **0.23** |
| - Within 30 days | 0.657 | 0.295 – 1.461 | **0.30** |
| CI, confidence interval. HR, hazards ratio; PNF, primary non-function. *Time to reLT. | | | |
